# The complete chloroplast genome of *Hylotelephium erythrostictum* (Miq.) H. Ohba (Crassulaceae)

**DOI:** 10.1080/23802359.2022.2036648

**Published:** 2022-02-09

**Authors:** Jing Chen, Hui Zhou, Wei Gong

**Affiliations:** a College of Life Science, Zhejiang Chinese Medical University, Hangzhou, PR China; b College of Life Sciences, South China Agricultural University, Guangzhou, PR China

**Keywords:** *Hylotelephium erythrostictum*, chloroplast genome, ornamental plant, Crassulaceae

## Abstract

The species *Hylotelephium erythrostictum* is well known as an ornamental plant in China. Here, we report the complete chloroplast genome (cp) of *H. erythrostictum* using the next-generation sequencing. It shows a total length of 151,707 bp with typical quadripartite structure. The genome is composed of the large single copy region (LSC) of 83,070 bp, the small single copy region (SSC) of 17,018 bp, and two separated inverted regions (IRs) of 25,793 bp, respectively. It contains 134 genes, including 80 protein-coding genes (PCGs), 8 rRNA genes, and 37 tRNA genes. The overall GC content of the cp genome is 37.7%. Maximum likelihood (ML) tree based on ten complete chloroplast genomes of Crassulaceae and one outgroup species suggested a monophyly formed by *H. erythrostictum* together with *Hylotelephium ewersii*, which demonstrate a comparably closer phylogenetic relationship. The complete chloroplast genome of this *H. erythrostictum* provides valuable information and further phylogenetic reconstruction of the Crassulaceae family.

The genus *Hylotelephium,* belonging to the family of Crassulaceae, shows a wide geographical distribution range in Euro-Asia and North America. *Hylotelephium erythrostictum* (Miq.) H. Ohba 1977 is a perennial succulent herb in this genus. This species has been widely used in urban greening and ecological landscaping in China due to its strong stress-resistance and highly ornamental value. In this study, we report the first complete chloroplast (cp) genome of *H. erythrostictum*, on the purpose of further phylogenetic reconstruction of the Crassulaceae family.

Before we carried out the project, we got ethical approval and permission from the Natural Reserve of Tianmu Mountain. Field studies have been carried out in accordance with guidelines and comply with local legislation. One individual was collected from Tianmu Mountain, Zhejiang Province (N23.08°, E113.22°). A specimen was deposited at the herbarium of College of Life Science, Zhejiang Chinese Medical University (Jing Chen; cj00123@zcmu.edu.cn) under the voucher number 20150720-051-021. Tender leaves were sampled and instantly put into the silica gel for drying and preservation (Jing Chen; cj00123@zcmu.edu.cn). Total genomic DNA was extracted by using a modified CTAB method (Doyle and Doyle 1987). The paired-end (2 × 150 bp) library was sequenced by Illumina PE150 at Novogene Co. Ltd (Beijing, China). A total of 2.19 Gb clean reads were obtained after removing low quality reads and adaptor sequences. Using GetOrganelle (Jin et al. 2020) and SPAdes (Bankevich et al. 2012), we assembled the complete cp genome of *H. erythrostictum* followed by manual adjustment and annotated it by the aid of Geneious prime 2019.2.1 (Kearse et al. 2012), using default parameters to predict protein-coding genes (PCGs) and rRNA genes. The tRNA genes were annotated on ARAGORN (Laslett and Canback 2004). The cp genomes of nine species of Crassulaceae were downloaded from GenBank database, including *Aeonium arboreum* (MW206792), *Cotyledon tomentosa* (MW206793), *Crassula perforata* (MW206794), *Graptopetalum amethystinum* (MW206795), *Hylotelephium ewersii* (MN794014), *Kalanchoe fedtschenkoi* (MW206796), *Kalanchoe tomentosa* (MN794319), *Rosularia alpestris* (MN794333), and *Sedum lineare* (MT755626). *Eucommia ulmoides* (KU204775) of Eucommiaceae was used as an outgroup. Totally, 11 cp genomes were aligned with online software MAFFT on CIPRES (https://www.phylo.org, Miller et al. 2015). Maximum likelihood (ML) analyses were performed by using RAxML-HPC version 8.2.10 on XSEDE (https://www.phylo.org) with 1000 bootstrap replicates and the substitution GTR + I+G model (Stamatakis 2014), the latter of which was determined by the Bayesian information criterion (BIC) in jModeltest version 2.1.10 (Darriba et al. 2012).

The *H. erythrostictum* cp genome has been deposited in GenBank (Accession No.: MZ519882). The total length is 151,707 bp with the typical quadripartite structure. It consists of a pair of inverted regions (IRs) of 25,793 bp separated by large single copy region (LSC) of 83,070 bp and small single copy region (SSC) of 17,018 bp, respectively. The overall GC content of the cp genome is 37.7%. The whole cp genome of *H. erythrostictum* contains 134 genes with 80 PCGs, 37 tRNA genes, and 8 rRNA genes. Among these genes, 60 PCGs and 22 tRNA genes are located in the LSC region, while 12 PCGs and one tRNA gene occur in the SSC region. All these eight rRNA genes are duplicated in the IR regions. IR regions contain six PCGs and seven tRNA genes, if counting only once. Among the annotated genes, a total of 15 genes contain one intron, which are *trn*K-UUU*, trn*L-UAA*, rps*16*, trn*V-UAC*, rpl*2*, ndh*B*, trn*G-GCC*, trn*I-GAU*, trn*A-UGC*, atp*F*, ndh*A*, rpo*C1*, pet*D*, pet*B, and *rpl*16, while three genes including *clp*P*, ycf*3, and *rps*12 possess two introns. The numbers and genes that contain one or two introns are exactly the same as *Sedum tricarpum*, which is largely due to the low variation based on cpDNA genome and the relatively close phylogenetic relationship between *H. erythrostictum* and *Sedum tricarpum* in the Crassulaceae family. A well supported phylogenetic tree is reconstructed, suggesting a monophyly formed by *H. erythrostictum* together with *H. ewersii* (Figure 1). The two species demonstrate a comparably closer phylogenetic relationship with the clade consisting of *Graptopetalum amethystinum, Sedum lineare, Rosularia alpestris,* and *Aeonium arboreum.*

**Figure 1.**
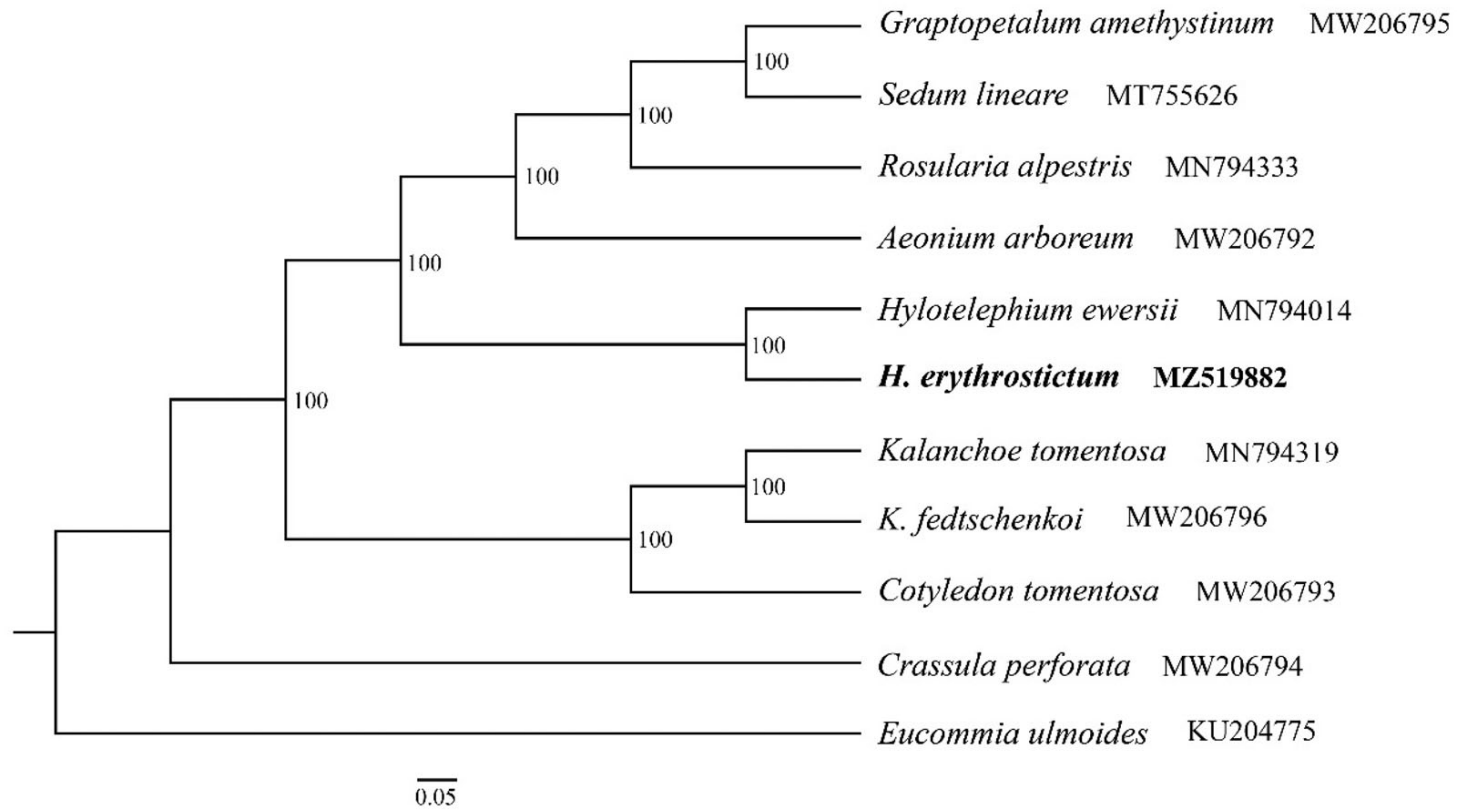
ML tree based on 10 complete chloroplast genomes of Crassulaceae and one outgroup species. Numbers at the nodes are bootstrap support values based on 1000 replicates. GenBank accession Numbers are listed beside the species. The species *H. erythrostictum* is highlighted in bold.

## Author contributions

Wei Gong and Jing Chen conceived and designed the project. Wei Gong and Hui Zhou analyzed and interpreted the data. Wei Gong and Jing Chen wrote the paper and revised it for intellectual content. All authors agree to be accountable for all aspects of the work.

## Data Availability

The complete chloroplast genome sequence data that support the findings of this study are openly available in GenBank of NCBI at [https://www.ncbi.nlm.nih.gov] (https://www.ncbi.nlm.nih.gov/) under the accession no. MZ519882. The associated BioProject, SRA, and Bio-Sample numbers are PRJNA749471, SRR15239599, and SAMN20371389, respectively.
